# Cis-trimethoxy resveratrol induces intrinsic apoptosis via prometaphase arrest and prolonged CDK1 activation pathway in human Jurkat T cells

**DOI:** 10.18632/oncotarget.23576

**Published:** 2017-12-22

**Authors:** Chae Eun Kim, Do Youn Jun, Jong-Sik Kim, Young Ho Kim

**Affiliations:** ^1^ Laboratory of Immunobiology, School of Life Science and Biotechnology, College of Natural Sciences, Kyungpook National University, Daegu 41566, Korea; ^2^ Leading-edge Research Center for Drug Discovery and Development for Diabetes and Metabolic Disease Bio-Medical Research Institute, Kyungpook National University Hospital, Daegu 702-201, Korea; ^3^ Department of Biological Sciences, Andong National University, Andong 36729, Korea

**Keywords:** CDK1-dependent mitochondrial apoptosis, mitotic spindle damage, phosphorylation of BCL-2 family proteins, prometaphase arrest, cis-trimethoxy resveratrol

## Abstract

Cis-trimethoxy resveratrol (cis-3M-RES) induced dose-dependent cytotoxicity and apoptotic DNA fragmentation in Jurkat T cell clones (JT/Neo); however, it induced only cytostasis in BCL-2-overexpressing cells (JT/BCL-2). Treatment with 0.25 μM cis-3M-RES induced G_2_/M arrest, BAK activation, Δψm loss, caspase-9 and caspase-3 activation, and poly (ADP-ribose) polymerase (PARP) cleavage in JT/Neo cells time-dependently but did not induce these events, except G_2_/M arrest, in JT/BCL-2 cells. Moreover, cis-3M-RES induced CDK1 activation, BCL-2 phosphorylation at Ser-70, MCL-1 phosphorylation at Ser-159/Thr-163, and BIM (BIM_EL_ and BIM_L_) phosphorylation irrespective of BCL-2 overexpression. Enforced G_1_/S arrest by using a G_1_/S blocker aphidicolin completely inhibited cis-3M-RES-induced apoptotic events. Cis-3M-RES-induced phosphorylation of BCL-2 family proteins and mitochondrial apoptotic events were suppressed by a validated CDK1 inhibitor RO3306. Immunofluorescence microscopy showed that cis-3M-RES induced mitotic spindle defects and prometaphase arrest. The rate of intracellular polymeric tubulin to monomeric tubulin decreased markedly by cis-3M-RES (0.1-1.0 μM). Wild-type Jurkat clone A3, FADD-deficient Jurkat clone I2.1, and caspase-8-deficient Jurkat clone I9.2 exhibited similar susceptibilities to the cytotoxicity of cis-3M-RES, excluding contribution of the extrinsic death receptor-dependent pathway to the apoptosis. IC_50_ values of cis-3M-RES against Jurkat E6.1, U937, HL-60, and HeLa cells were 0.07-0.17 μM, whereas those against unstimulated human peripheral T cells and phytohaemagglutinin A-stimulated peripheral T cells were >10.0 and 0.23 μM, respectively. These results indicate that the antitumor activity of cis-3M-RES is mediated by microtubule damage, and subsequent prometaphase arrest and prolonged CDK1 activation that cause BAK-mediated mitochondrial apoptosis, and suggest that cis-3M-RES is a promising agent to treat leukemia.

## INTRODUCTION

Resveratrol (3,5,4′-trihydroxystilbene) is a naturally produced polyphenolic compound belonging to the phytoalexin group that is produced by plants under stress. Resveratrol exerts multiple beneficial biological effects on human health, including anti-inflammatory, antioxidant, anti-aging, anti-obesity, cardioprotective, and anticancer effects [1, 2]. Recent studies have focused on the anticancer effect of resveratrol because it exerts potential chemopreventive effects, induces cell cycle arrest, and possesses apoptogenic properties. Although numerous data of anticancer properties of resveratrol are available from *in vitro* studies on many tumor cell lines, its action shows poor efficacy in *in vivo* trials possibly due to low oral bioavailability, rapid metabolism, and low tissue concentration [2–5]. In this context, several trials have assessed a series of resveratrol analogues and have evaluated their cytostatic and cytotoxic activities to improve the anticancer activity of resveratrol [1, 2, 6–9]. Recently, cis-3,5,4′-trimethoxy resveratrol (cis-3M-RES), a naturally occurring resveratrol analogue, has been chemically synthesized and has been examined as a more promising chemopreventive agent which exerts 100-fold higher cytotoxicity against several human tumors than resveratrol [6, 9].

Cis-3M-RES exerts cytotoxic effects on human colon adenocarcinoma Caco-2 cells at pharmacological concentrations through induction of mitotic arrest by interfering tubulin polymerization (IC_50_ = 4 μM), and apoptotic DNA fragmentation [6, 9]. Although previous studies indicate that cis-3M-RES induces mitotic arrest and apoptosis, limited information is available on the correlation between cell cycle arrest and apoptosis induction in cis-3M-RES-treated tumor cells. Molecular mechanisms underlying the impact of cis-3M-RES on cellular microtubule network and apoptotic regulatory system should be studied further to clarify whether the antitumor effects of cis-3M-RES are confined to tumor cells or extend to normal cells. Results of these studies will expand our understanding of the efficacy of cis-3M-RES as a chemopreventive agent for cancer managements.

The efficacy of chemotherapy in inducing tumor regression mainly depends on the anti-proliferative and/or pro-apoptotic effects of chemotherapeutic drugs on tumor cells [10]. Because apoptosis of tumor cells leads to their destruction into apoptotic bodies that are cleared by phagocytic cells without causing a local inflammatory response, apoptosis induction is proposed as an efficient mechanism for removing malignant tumor cells after chemotherapy [11, 12]. Three cell death signaling pathways are suggested to be involved in chemotherapeutic drug-induced tumor cell apoptosis, namely, extrinsic death receptor-dependent pathway [13], intrinsic mitochondria-dependent pathway [14], and intrinsic endoplasmic reticulum stress-mediated pathway [15]. The intrinsic mitochondria-dependent pathway is the most frequent pathway associated with tumor cell apoptosis induced by chemotherapeutic drugs, such as DNA-damaging agents (DDAs) and microtubule-damaging agents (MDAs) [16].

Recently, we decided to take advantage of BCL-2 overexpression, which blocks the intrinsic mitochondria-dependent apoptotic pathway [17], to determine the association between cis-3M-RES-induced mitotic cell cycle arrest and apoptotic cell death. Previously, we used BCL-2 overexpression to elucidate the involvement of microtubule damage-mediated G_2_/M arrest in microtubule damage-mediated apoptosis of human acute leukemia Jurkat T cells, in which the apoptotic pathways occurring upstream of BCL-2-sensitive mitochondrial apoptotic events are more prominently detected when the mitochondrial apoptotic pathway is blocked by BCL-2 overexpression [18–20]. In this study, we compared cis-3M-RES-induced cell cycle arrest and apoptotic signaling pathway in Jurkat T cell clones stably transfected with an empty vector (JT/Neo cells) or the *BCL-2* expression vector (JT/BCL-2 cells). To examine whether cis-3M-RES-induced cell cycle arrest is required for apoptosis induction, we investigated the effect of aphidicolin (APC), which arrests cell cycle progression at the G_1_/S border [21], on cis-3M-RES-induced apoptosis. Additionally, we compared the IC_50_ values of cis-3M-RES against human leukemia cells (Jurkat, U937, and HL-60), human cervical carcinoma HeLa, and normal human peripheral T cells to examine whether normal cells are more refractory to the cytotoxicity of cis-3M-RES than malignant tumor cells.

## RESULTS

### Apoptogenic effect of cis-3M-RES on human Jurkat T cell clones JT/Neo and JT/BCL-2

**Figure 1:**
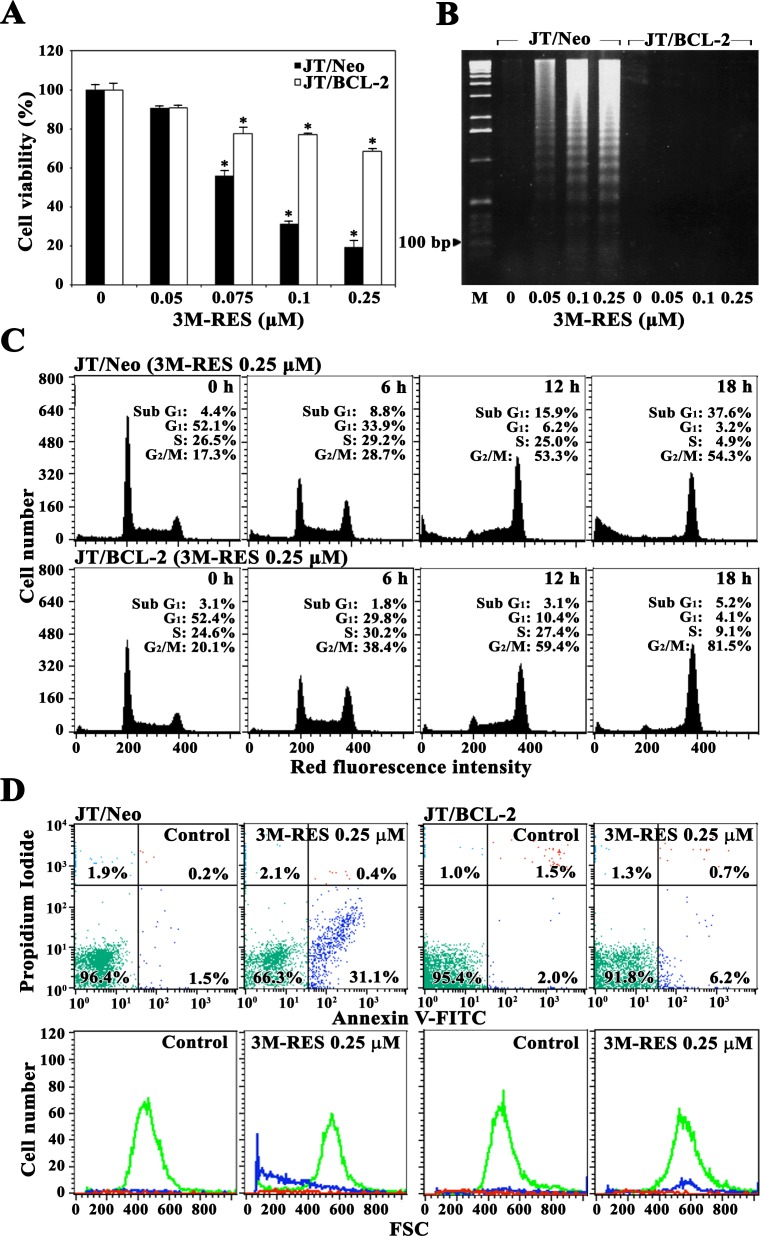
Effect of cis-3M-RES on cell viability, apoptotic DNA fragmentation, cell cycle distribution, and apoptotic cell death in Jurkat T cell clones transfected with an empty vector (JT/Neo cells) or the *BCL*-2-expression vector (JT/BCL-2 cells) **(A)** Cell viability was determined by incubating each cell type (5 × 10^4^ cells/well) with the indicated concentrations of cis-3M-RES in a 96-well plate for 36 h and the final 4 h was incubated with MTT. Each value is expressed as the mean ± SEM (n = 3; three replicates per independent experiment). ^*^P < 0.05 compared with control. **(B)** Equivalent cultures were prepared, and the cells were collected to analyze apoptotic DNA fragmentation by performing Triton X-100 lysis with 1.2% agarose gel electrophoresis. **(C-D)** Cell cycle distribution and apoptosis/necrosis were determined by performing flow cytometric analysis with PI staining and FITC-Annexin V/PI double staining, respectively. A representative result is shown; two additional experiments yielded similar results.

To examine whether the BCL-2-sensitive mitochondrial apoptotic pathway is the key mediator of cytotoxicity induced by cis-3M-RES (0.05-0.25 μM), we compared the cytotoxic effect of cis-3M-RES on JT/Neo and JT/BCL-2 cells. Results of 3-(4,5-dimethylthiazol-2-yl)-2,5-diphenyltetrazolium bromide (MTT) assay showed that the viabilities of JT/Neo cells exposed to 0.05, 0.075, 0.1, and 0.25 μM cis-3M-RES for 36 h were 90.7%, 55.8%, 31.3%, and 19.3%, respectively, with an IC_50_ value of 0.08 μM, whereas those of JT/BCL-2 cells were 90.9%, 77.6%, 77.0%, and 68.6%, respectively, with an IC_50_ value of >10.0 μM (Figure 1A). Furthermore, we observed that treatment with 0.05-0.25 μM cis-3M-RES induced apoptotic DNA fragmentation in JT/Neo cells but not in JT/BCL-2 cells in a concentration-dependent manner (Figure 1B). Apoptotic sub-G_1_ cells were not detected in JT/Neo cells until 6 h after 0.25 μM cis-3M-RES treatment but were detected in a time-dependent manner between 12 and 18 h after 0.25μM cis-3M-RES treatment (Figure 1C). However, the proportion of apoptotic sub-G_1_ cells did not increase in JT/BCL-2 cells after cis-3M-RES treatment. Under these conditions, the proportion of G_2_/M cells significantly increased in a time-dependent manner in both JT/Neo and JT/BCL-2 cells but that of G_1_-S cells did not increase in both cell types after cis-3M-RES treatment.

Analysis of JT/Neo cells treated with 0.25 μM cis-3M-RES for 18 h by performing FITC-Annexin V and PI staining showed that the number of early apoptotic cells, which were stained only with FITC-Annexin V, increased, and the numbers of late apoptotic cells, which were stained with both FITC-Annexin V and PI, and of necrotic cells, which were stained with only PI, were negligible (Figure 1D). However, cis-3M-RES-induced increase in the number of early apoptotic cells was not observed in JT/BCL-2 cells.

These results indicate that cis-3M-RES exerts a cytostatic effect by inducing G_2_/M arrest of the cell cycle, and BCL2-sensitive apoptotic cell death without inducing necrosis. Moreover, these results indicate that G_2_/M arrest is induced by a mechanism that is independent of the anti-apoptotic effect of BCL-2.

### Comparison of mitochondrial membrane potential loss, BAK activation, CDK1 activation, BCL-2 phosphorylation, MCL-1 phosphorylation, BIM phosphorylation, and caspase cascade activation in cis-3M-RES-treated JT/Neo and JT/BCL-2 cells

**Figure 2:**
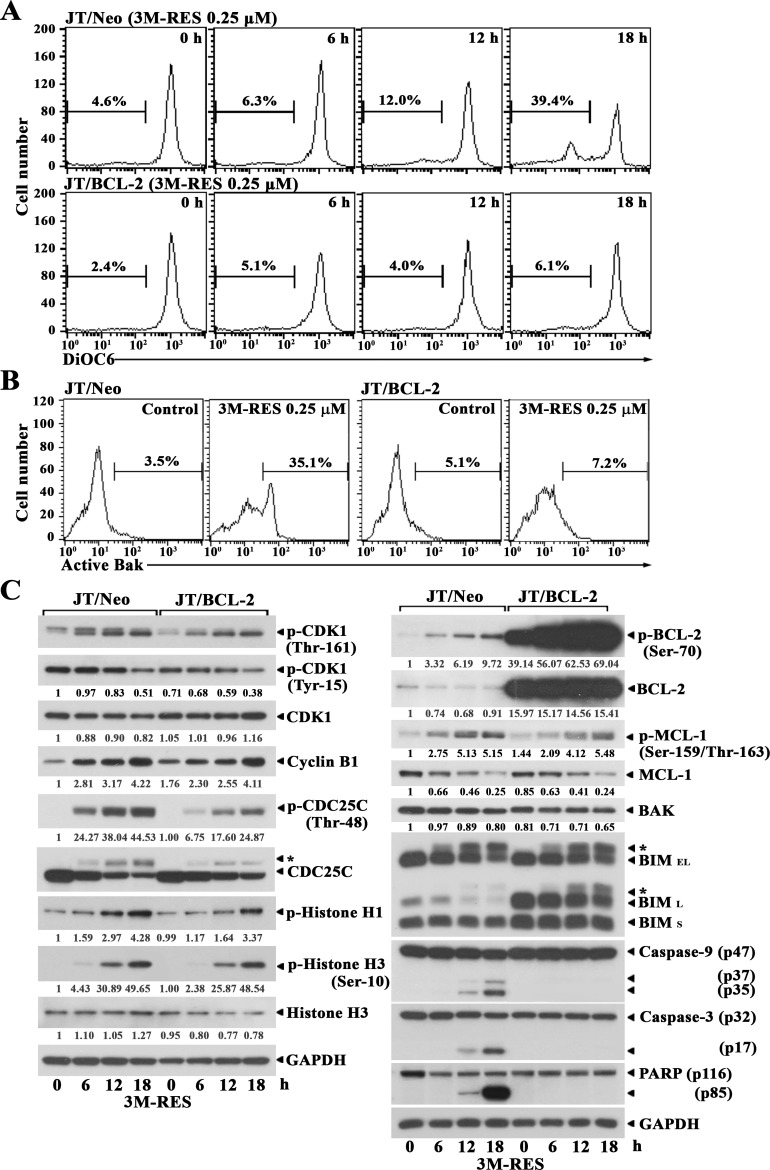
Flow cytometric analysis of Δψm loss and BAK activation, and western blot analysis of phosphorylated CDK1 (Thr-161 and Tyr-15), CDK1, cyclin B1, phosphorylated CDC25C (Thr-48), CDC25C, phosphorylated histone H1, phosphorylated histone H3 (Ser-10), histone H3, GAPDH, phosphorylated BCL-2 (Ser-70), BCL-2, phosphorylated MCL-1 (Ser159/Thr-163), MCL-1, BAK, BIM (BIM_EL_ and BIM_L_), caspase-9 and caspase-3 activation, PARP cleavage, and GAPDH in cis-3M-RES-treated JT/Neo and JT/BCL-2 cells **(A-B)** Each cell type (5 × 10^5^/ml) was treated with 0.25 μM cis-3M-RES for indicted time periods, and subjected to flow cytometric analysis of Δψm loss and BAK activation as described in Materials and Methods. **(C)** Equivalent cultures were prepared for analysis of total cell lysates by western blotting as described in Materials and Methods. Symbol: ← ^*^, phosphorylated form of CDC25C and BIM. A representative result is shown; two additional experiments yielded similar results.

To examine whether cis-3M-RES-induced apoptosis is mediated by the BCL-2-sensitive mitochondria-dependent apoptotic pathway, which is known to be frequently involved in chemotherapeutic drug-induced apoptosis [14, 22], we measured changes in mitochondrial membrane potential (Δψm) in JT/Neo cells treated with 0.25 μM cis-3M-RES for different time periods by performing flow cytometry with 3,3′-dihexyloxacarbocyanine iodide (DiOC_6_) staining. Although JT/Neo cells treated with 0.25 μM cis-3M-RES for 6 h showed negligible Δψm loss, cells treated with 0.25 μM cis-3M-RES for 12 h and 18 h showed Δψm loss of 12.0% and 39.4%, respectively (Figure 2A). However, JT/BCL-2 cells treated with cis-3M-RES did not show Δψm loss. To further examine the involvement of the mitochondria-dependent apoptotic pathway in cis-3M-RES-induced apoptosis of Jurkat T cells, we analyzed BAK activation by performing flow cytometry with conformation-specific anti-BAK (Ab-1) antibody [23]. As shown in Figure 2B, BAK activation was detected in JT/Neo cells but not in JT/BCL-2 cells treated with 0.25 μM cis-3M-RES, indicating that BAK activation, which was blocked by BCL-2, was involved in cis-3M-RES-induced Δψm loss. Because BAK-mediated Δψm loss is one of the initial intracellular changes associated with mitochondria-dependent apoptosis [24], the above results indicate an association between mitochondrial apoptotic events and cis-3M-RES-induced apoptosis in Jurkat T cells.

Two molecular mechanisms are implicated in cytotoxic agent-induced G_2_/M arrest accompanying the apoptosis of tumor cells, namely, G_2_ checkpoint pathway [25] and mitotic spindle assembly checkpoint pathway [26]. When cells are arrested at the G_2_ checkpoint, the mitotic kinase CDK1/cyclin B remains in an inactive state because of the failure of CDC25-mediated dephosphorylation of inhibitory phosphates at Thr-14/Tyr-15. In contrast, when cells were arrested at the mitotic spindle assembly checkpoint, CDK1/cyclin B is maintained in a completely active state through CDK-activating kinase (CAK)-mediated activating phosphorylation at Thr-161 and CDC25-mediated removal of inhibitory phosphorylation at Thr-14/Tyr-15. To examine whether CDK1 is present in the active state during cis-3M-RES-mediated G_2_/M cell cycle arrest, we compared the phosphorylation status of CDK1 at Tyr-15 and Thr-161, which is critical for its activation, in cis-3M-RES-treated JT/Neo cells and JT/BCL-2 cells by performing western blotting. The level of CDK1 phosphorylated at Tyr-15 decreased, the level of CDK1 phosphorylated at Thr-161 increased, the expression level of cyclin B1 increased, and the total expression of CDK1 remained relatively constant in cis-3M-RES-treated JT/Neo cells and JT/BCL-2 cells (Figure 2C). Moreover, histone H1 phosphorylation, which is catalyzed by CDK1 in the G_2_/M phase [27], and histone H3 phosphorylation at Ser-10 by ARK2, which is increased by CDK1 during the G_2_/M phase [28, 29], also increased in both cell types. CDC25C phosphorylation at Thr-48, which is necessary for CDK1 dephosphorylation at Tyr-15 also increased in both cell types. These results indicate that CDK1 is activated and its enzymatic activity is sustained during cis-3M-RES-induced mitotic arrest. In addition, BCL-2 phosphorylation at Ser-70, MCL-1 phosphorylation at Ser-159 and/or Thr-163, and BIM (BIM_EL_ and BIM_L_) phosphorylation, as evidenced by their phosphorylation-induced reduction in mobility during SDS-polyacrylamide gel electrophoresis, increased in both JT/Neo and JT/BCL-2 cells after cis-3M-RES treatment. Although BCL-2 and BAK expression levels remained relatively constant after cis-3M-RES treatment, the MCL-1 expression level markedly decreased along with the enhancement in its phosphorylaton level. In accordance with cis-3M-RES-induced BAK activation in JT/Neo cells, caspase-9 activation that proceeded through proteolytic cleavage of the inactive proenzyme (47 kDa) to its active forms (37/35 kDa), caspase-3 activation, through the proteolytic degradation of the 32-kDa proenzyme to its 17-kDa activated form, and the cleavage of poly (ADP-ribose) polymerase (PARP) were detected. However, cis-3M-RES-induced BAK activation, caspase-9 and caspase-3 activation, and PARP cleavage were completely abrogated in JT/BCL-2 cells overexpressing BCL-2.

These results indicate that the cis-3M-RES-induced apoptosis of Jurkat T cells involves G_2_/M arrest; CDK1 activation; BCL-2, MCL-1 and BIM phosphorylation; BAK activation; Δψm loss, caspase-9 and caspase-3 activation, and PARP cleavage. These results also indicate that CDK1 activation and BCL-2, MCL-1 and BIM phosphorylation occur upstream of BCL-2-preventable BAK activation and caspase cascade activation in cis-3M-RES-treated JT/Neo cells.

### Effect of APC on the cis-3M-RES-induced G_2_/M-arrest, CDK1 activation, phosphorylation of BCL-2, MCL-1, and BIM, mitochondrial damage, and subsequent activation of caspase-9

**Figure 3:**
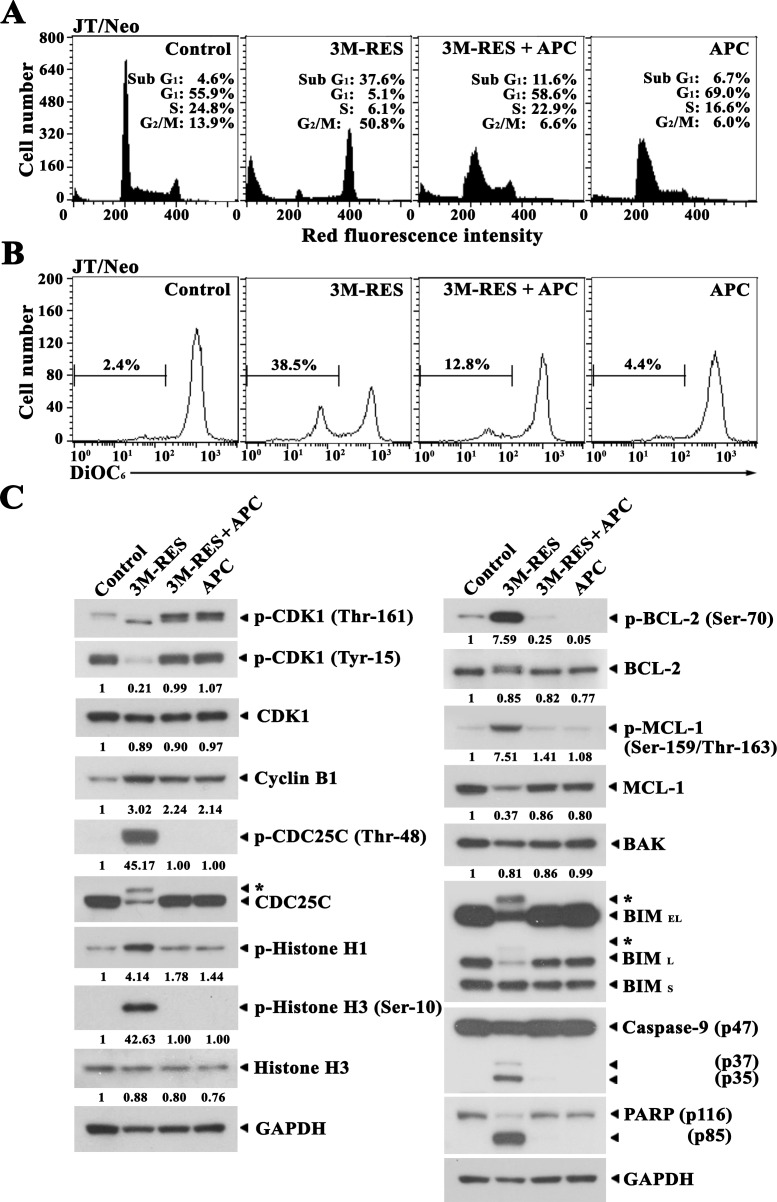
Inhibitory effect of APC on cis-3M-RES-induced G_2_/M arrest, Δψm loss, CDK1 phosphorylation (Thr-161 and Tyr-15), cyclin B1, histone H1 phosphorylation, CDC25C phosphorylation (Thr-48), BCL-2 phosphorylation (Ser-70), MCL-1 phosphorylation (Ser-159/Thr-163), MCL-1, BIM (BIM_EL_ and BIM_L_), caspase-9 activation, PARP cleavage, and GAPDH in JT/Neo cells **(A-B)** After JT/Neo cells (5 × 10^5^/ml) were treated with 0.25 μM cis-3M-RES, 0.25 μM cis-3M-RES and 0.5 μM APC, or 0.5 μM APC for 20 h, the cells were subjected to flow cytometric analysis of cell cycle distribution and Δψm loss. **(C)** Equivalent cultures were prepared for analysis of total cell lysates by western blotting as described in Materials and Methods. Symbol: ← ^*^, phosphorylated form of CDC25C and BIM. A representative result is shown; two additional experiments yielded similar results.

To further examine the dependency of cis-3M-RES-induced apoptotic events on G_2_/M cell cycle arrest, we investigated the effect of APC, which blocks the cell cycle at the G_1_/S border by inhibiting DNA polymerase α [21], on the cis-3M-RES-induced apoptotic events. When JT/Neo cells were treated with 0.5 μM APC for 20 h, 69.0% cells were in the G_1_ phase, 16.6% cells were in the S phase, 6.0% cells were in the G_2_/M phase, and 6.7% cells were in the apoptotic sub-G_1_ phase, suggesting that majority of the cells were arrested at the G_1_/S border (Figure 3A). When JT/Neo cells were treated with 0.25 μM cis-3M-RES for 20 h, 37.6% cells were in the apoptotic sub-G_1_ phase and 5.1%, 6.1%, and 50.8% cells were in the G_1_, S, and G_2_/M phases, respectively. APC treatment almost completely abrogated cis-3M-RES-induced G_2_/M arrest and apoptotic sub-G_1_ peak. Moreover, APC treatment significantly suppressed cis-3M-RES-induced Δψm loss (Figure 3B). These results indicate that cis-3M-RES did not induce mitotic arrest, Δψm loss, and apoptosis in JT/Neo cells concomitantly treated with APC, which caused majority of cells to accumulate at the G_1_/S border.

Results of western blot analyses showed that cis-3M-RES-induced BCL-2 phosphorylation at Ser-70, MCL-1 phosphorylation at Ser-159 and/or Thr-163, BIM phosphorylation, histone H1 phosphorylation, histone H3 phosphorylation at Ser-10, capase-9 activation, and PARP cleavage were not or were negligibly detected in the presence of APC (Figure 3C). Moreover, cis-3M-RES-induced CDK1 dephosphorylation at Tyr-15, CDK1 phosphorylation at Thr-161, and CDC25C phosphorylation at Thr-48, which are required for CDK1 activation during G_2_/M transition of the cell cycle [30, 31], histone H1 phosphorylation, and histone H3 phosphorylation were not detected in the presence of APC.

These results indicate that cis-3M-RES-induced apoptotic events, including CDK1 activation; BCL-2, MCL-1 and BIM phosphorylation; Δψm loss; caspase-9 activation; and PARP cleavage, occurred as a consequence of mitotic arrest.

### Effect of the CDK1 inhibitor RO3306 and the pan-caspase inhibitor z-VAD-fmk on cis-3M-RES-induced apoptotic events

Previously, it has been reported that several MDAs including taxol can induce CDK1-mediated serine phosphorylation of BCL-2 and MCL-1, which results in abrogation of their anti-apoptotic function, suggesting a pro-apoptotic role of CDK1 as the BCL-2 and MCL-1 kinase [32, 33]. Our studies have also shown that Jurkat T cells treated with MDAs including NOC and 2-MeO-E_2_ commonly undergo mitotic arrest-caused prolonged CDK1 activation and resultant phosphorylation of BCL-2, MCL-1, and BIM, and onset of mitochondria-dependent apoptosis by sequentially triggering BAK activation, Δψm loss, and caspase cascade activation [18–20].

**Figure 4:**
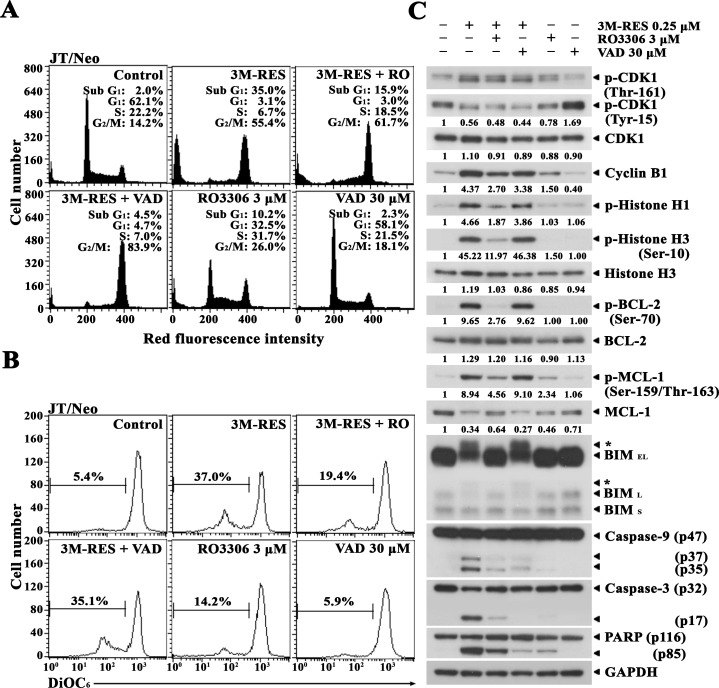
Differential suppressive effects of the CDK1 inhibitor RO3306 and the pan-caspase inhibitor z-VAD-fmk on cis-3M-RES-induced apoptotic events in JT/Neo cells **(A-B)** After JT/Neo cells were treated with 0.25 μM cis-3M-RES in the absence or in the presence of 3 μM RO3306, or 30 μM z-VAD-fmk for 20 h, the cells were collected for flow cytometric analysis of cell cycle distribution and Δψm loss. **(C)** Equivalent cultures were prepared for analysis of total cell lysates by western blotting as described in Materials and Methods. Symbol: ← ^*^, phosphorylated form of CDC25C and BIM. A representative study is shown; two additional experiments yielded similar results.

To verify that the cis-3M-RES-induced CDK1 activation is the upstream event of phosphorylation of BCL-2, MCL-1 and BIM, Δψm loss, and caspase activation, and to confirm further the requirement of the caspase activation pathway for cis-3M-RES-induced apoptosis, we examined the effect of the CDK1 inhibitor RO3306 [34] and the pan-caspase inhibitor z-VAD-fmk [35] on cis-3M-RES-induced apoptotic events in JT/Neo cells. After treatment of JT/Neo cells with 0.25 μM cis-3M-RES for 20 h, the apoptotic sub-G_1_ peak reached 35.0%; however, it declined to 15.9% by concomitant treatment with 0.25 μM cis-3M-RES and 3 μM RO3306, indicating a remarkable suppression of the sub-G_1_ peak by ∼54.6% (Figure 4A). The cis-3M-RES-induced Δψm loss, which appeared to increase by 37.0% in cis-3M-RES-treted JT/Neo cells, was reduced to 14.2% in the presence of 3 μM RO3306 (Figure 4B). Under these conditions, cis-3M-RES-induced phosphorylation of histone H1, which is catalyzed by the mitotic CDK1 [27], was abrogated by RO3306, indicating an RO3306-mediated effective inhibition of CDK1 (Figure 4C). When the cis-3M-RES-induced histone H1 phosphorylation was prevented by RO3306, BCL-2, MCL-1, and BIM phosphorylation, caspase-9 and caspase-3 activation, and PARP cleavage were reduced to a barely detectable or undetectable level. These results indicate that cis-3M-RES-induced phosphorylation of BCL-2, MCL-1, and BIM, which led to mitochondria-dependent caspase cascade activation, was mediated by CDK1. Although the presence of 30 μM z-VAD-fmk completely prevented cis-3M-RES-induced apoptotic sub-G_1_ peak, it failed to prevent the G_2_/M-arrest and Δψm loss. Western blot analysis also revealed that cis-3M-RES-induced activation of caspase-9 and caspase-3, and PARP cleavage were significantly reduced by z-VAD-fmk, whereas cis-3M-RES-induced BCL-2 phosphorylation at Ser-70, MCL-1 phosphorylation at Ser-159 and/or Thr-163, and BIM (BIM_EL_ and BIM_L_) phosphorylation were sustained. These results confirm that cis-3M-RES-induced G_2_/M-arrest, phosphorylation of BCL-2, MCL-1, and BIM, and Δψm loss occurred upstream of the caspase cascade activation.

Consequently, these results demonstrate that the activation of caspase cascade including caspase-9 and caspase-3 was a prerequisite for cis-3M-RES-induced apoptosis, and that cis-3M-RES-induced G_2_/M-arrest and mitotic CDK1-dependent phosphorylation of BCL-2, MCL-1, and BIM, which were refractory to the inhibitory action of the pan-caspase inhibitor z-VAD-fmk, were upstream events of mitochondrial damage and resultant caspase cascade activation.

### Effect of cis-3M-RES on cellular microtubule network in JT/Neo and JT/BCL-2 cells

**Figure 5:**
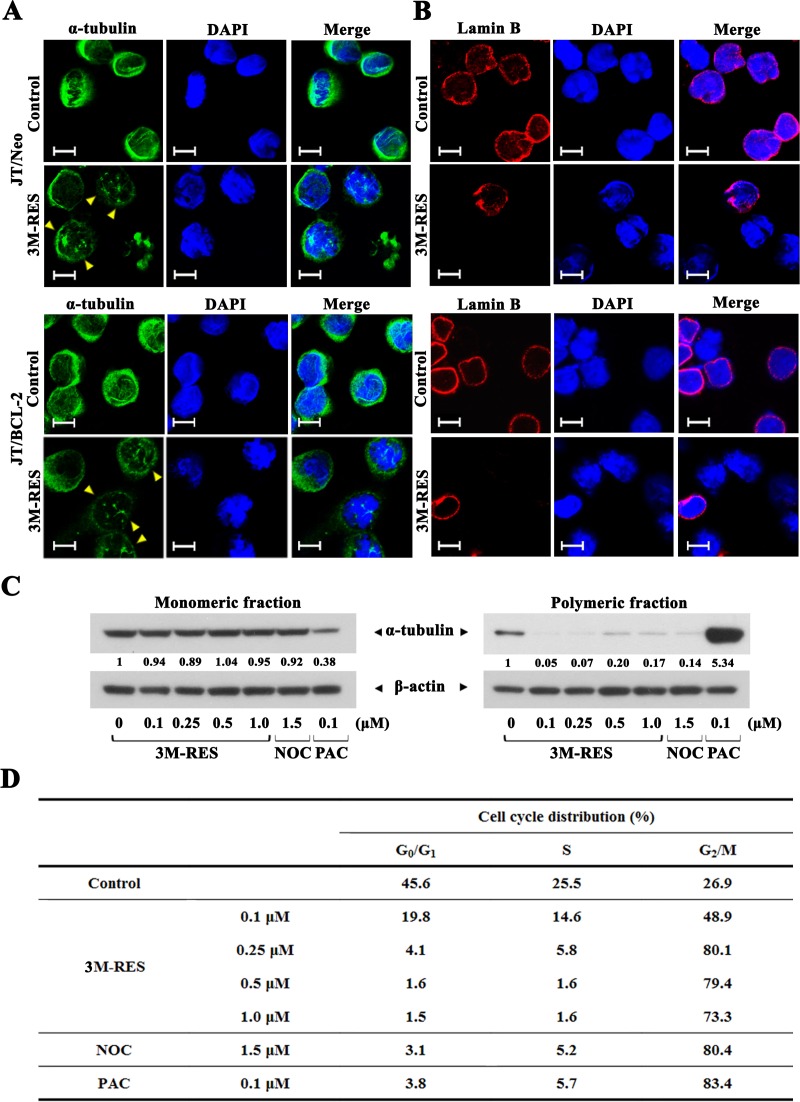
Cis-3M-RES-induced alterations in the organization of microtubules (α-tubulin) and the nuclear envelope (Lamin B) in JT/Neo and JT/BCL-2 cells, and cis-3M-RES-induced changes in the ratio of intracellular monomeric to polymeric tubulin, and cell cycle distribution **(A-B)** After JT/Neo and JT/BCL-2 cells were treated with 0.25 μM cis-3M-RES for 20 h, the cells were made to adhere to glass cover slips and fixed using cold methanol for 3 min, permeabilized, and blocked using 10% goat serum or 10% donkey serum for 30 min. The cells were then incubated overnight at 4 °C with mouse monoclonal anti-α-tubulin (dilution, 1:2500) or goat polyclonal anti-lamin B (dilution, 1:200) antibody. For detection, the cells were treated with Alexa Fluor 488-labeled goat anti-mouse IgG or Alexa Fluor 568-labeled donkey anti-goat IgG. To label the nuclei, the cells were stained with DAPI. Images were captured using a Carl Zeiss MicroImaging Confocal Laser Scanning Microscope; scale bar = 10 μm. **(C-D)** Western blot analyses to detect intracellular monomeric and polymeric α-tubulin, and flow cytometric analysis of cell cycle distribution of JT/BCL-2 treated with 0.25 μM cis-3M-RES for 20 h were performed as described in Materials and Methods. Symbol: yellow arrowhead, aberrant bipolar array of microtubules. A representative result is shown; and two additional experiments yielded similar results.

To examine whether cis-3M-RES-induced G_2_/M arrest was associated with mitotic spindle defect, we investigated the effect of cis-3M-RES on microtubule network organization in JT/Neo cells and JT/BCL-2 cells by immunofluorescence microscopy with anti-α-tubulin antibody. Majority of exponentially growing JT/Neo cells and JT/BCL-2 cells showed normal arrangement of the microtubule network. However, treatment with 0.25 μM cis-3M-RES for 20 h resulted in the formation of an aberrant bipolar array of microtubules in both cell types (Figure 5A). Typical apoptotic bodies were observed in JT/Neo cells but not in JT/BCL-2 cells treated with cis-3M-RES. In addition, 4',6-diamidino-2-phenylindole (DAPI) staining showed that most chromosomes in both cell types treated with cis-3M-RES were not properly aligned at the equator of the mitotic spindle. Analysis of nuclear envelop breakdown by performing immunofluorescence microscopy with anti-lamin B antibody showed absence of the nuclear envelope in cis-3M-RES-treated cells showing mitotic arrest (Figure 5B). Prometaphase begins with the breakdown of the nuclear envelope and continues until sister chromatids attached to the mitotic spindle are aligned at the center of the spindle [36]. The above results indicate that cis-3M-RES-induced mitotic arrest is induced by the blockade of the prometaphase because of defects in the mitotic spindle and because of the failure of chromosome congregation at the equatorial plate.

To elucidate whether cis-3M-RES affected microtubule polymerization, we analyzed the proportion of polymerized cellular tubulin in JT/BCL-2 cells treated with 0.1, 0.25, 0.5, or 1.0 μM cis-3M-RES for 20 h. Cells treated with microtubule-depolymerizing drug nocodazole (1.5 μM) [37] and microtubule-polymerizing drug paclitaxel (0.1 μM) [38] were used as controls. The ratio of polymeric to monomeric tubulin decreased after nocodazole treatment but increased after paclitaxel treatment (Figure 5C). Treatment of JT/BCL-2 cells with 0.1-1.0 μM cis-3M-RES decreased the ratio of polymeric to monomeric tubulin. Although treatment with nocodazole, paclitaxel, and cis-3M-RES exerted different effects on cellular microtubule polymerization, JT/BCL-2 cells separately treated with these drugs showed G_2_/M arrest (Figure 5D). These results indicate that cis-3M-RES-induced prometaphase arrest, which was detected in Jurkat T cells irrespective of BCL-2 overexpression, is induced by the reduction in intracellular tubulin polymerization.

### Comparison of the cytotoxic effect of cis-3M-RES on wild-type Jurkat T cell clone A3, FADD-deficient Jurkat T cell clone I2.1, and caspase-8-deficient Jurkat T cell clone I9.2

**Figure 6:**
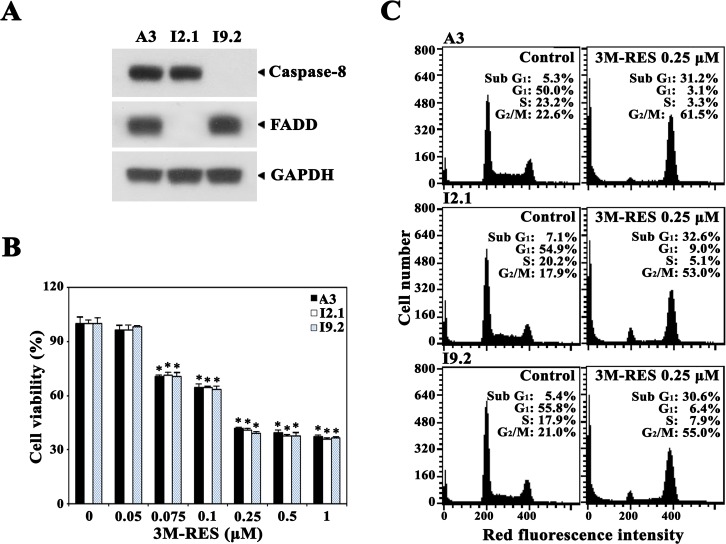
Effect of cis-3M-RES on cell viability and cell cycle distribution in FADD- and caspase-8-positive wild-type Jurkat T cells (clone A3), FADD-deficient Jurkat T cells (clone I2.1), and caspase-8-deficient Jurkat T cells (clone I9.2) **(A)** Expression of FADD and caspase-8 in A3, I2.1 and I9.2 cells were confirmed by western blot analysis as described in Material and Methods. **(B)** To measure cytotoxic effect of 0.25 μM cis-3M-RES on A3, I2.1, and I9.2 cells, individual cells (5 × 10^4^ cells/well) were incubated with 0.05-1 μM cis-3M-RES in a 96-well plate for 36 h and the final 4 h was incubated with MTT to assess cell viability. Each value is expressed as mean ± SEM (n = 3 with three replicates per independent experiment). ^*^P < 0.05 compared with control. **(C)** Apoptotic changes in cell cycle distribution of A3, I2.1, and I9.2 cells following treatment with 0.25 μM cis-3M-RES for 20 h was determined by flow cytometry as described in Materials and Methods.

Upregulation of FasL and/or Fas expression is suggested to be a potential mechanism underlying antineoplastic drug-induced apoptosis [39, 40]. To examine the involvement of death receptor (DR)/DR ligand system in cis-3M-RES-induced apoptosis, we compared the cytotoxic effect of cis-3M-RES on wild-type Jurkat T cell clone A3 with that on Fas-associated death domain (FADD)-deficient Jurkat T cell clone I2.1 and caspase-8-deficient Jurkat T cell clone I9.2, which are refractory to Fas-mediated apoptosis [41]. As shown in Figure 6A, results of western blot analysis confirmed that I2.1 and I9.2 cells did not express FADD and caspase-8, respectively. Results of MTT assay showed that FADD-deficient I2.1 cells, caspase-8-deficient I9.2 cells, and wild-type A3 cells treated with 0.05-1.0 μM cis-3M-RES for 20 h in 96-well plates exhibited similar sensitivities to cis-3M-RES-induced cytotoxicity (Figure 6B). Flow cytometric analysis also showed that all Jurkat T cell clones were similarly sensitive to cis-3M-RES-induced apoptogenicity (Figure 6C). These results confirm that cis-3M-RES-induced apoptosis of Jurkat T cells is mediated by the intrinsic mitochondrial apoptotic pathway and not by the extrinsic DR-dependent apoptotic pathway, which mediates the death signaling through FADD and caspase-8.

### Cytotoxic effect of cis-3M-RES on human peripheral T cells and malignant tumor cells

**Figure 7:**
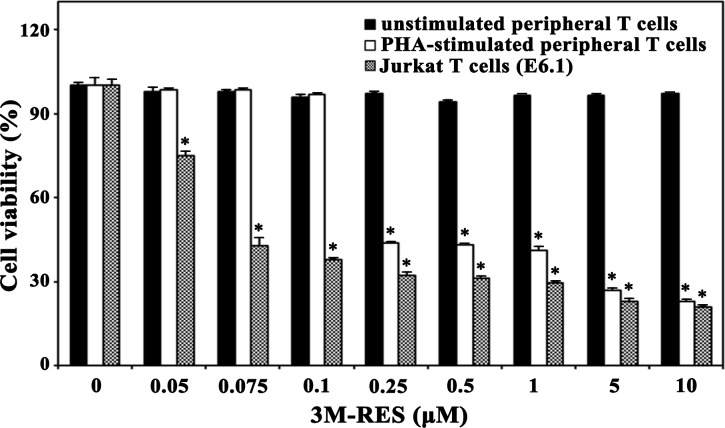
Effect of cis-3M-RES on unstimulated human T cells, IL-2-dependent proliferation of PHA-stimulated T cells, and proliferation of Jurkat T cells (E6.1) Normal human peripheral T cells (2 × 10^5^ cells/well) were incubated with 0.05-10 μM cis-3M-RES in a 96-well plate for 36 h, and the final 4 h was incubated with MTT solution to assess cell viability. To induce IL-2-dependent proliferation of activated T cells, human peripheral T cells were stimulated with PHA (1.0 μg/ml) for 60 h, and then the stimulated T cells were harvested and incubated with 0.05-10 μM cis-3M-RES (1 × 10^5^ cells/well) and 25 U/ml of recombinant human IL-2 in 96-well plates. For treatment of Jurkat E6.1 cells with cis-3M-RES, the cell density was 5 × 10^4^ cells/well. Each value is expressed as mean ± SEM (n = 3 with three replicates per independent experiment). ^*^P < 0.05 compared with control.

Because cis-3M-RES showed potent cytotoxicity against malignant Jurkat T cells, we examined whether cis-3M-RES is less cytotoxic toward normal T cells. We investigated the cytotoxic effects of cis-3M-RES on the viability of unstimulated human peripheral T cells or the interleukin-2 (IL-2)-dependent proliferation of activated T cells, which were obtained by stimulating human peripheral T cells with 1.0 μg/ml phytohaemagglutinin A (PHA) for 60 h. Results of MTT assay showed that treatment with 0.05-10.0 μM cis-3M-RES did not markedly affect the viability of unstimulated peripheral T cells (Figure 7). Under the same conditions, the IL-2-dependent proliferation of activated T cells was not affected at concentrations up to 0.1 μM and decreased to a basal level at cis-3M-RES concentration of >0.25 μM. However, the viability of malignant Jurkat T cell clone E6.1 decreased to 74.9%, 42.7%, and 37.9% after treatment with 0.05, 0.75, and 0.1 μM, respectively. The sensitivities of human leukemia cells (U937 and HL-60) and human cervical carcinoma HeLa cells to the cytotoxicity of cis-3M-RES were also investigated. As shown in Table 1, the IC_50_ values of cis-3M-RES for unstimulated human peripheral T cells and IL-2-dependent proliferation of PHA-stimulated peripheral T cells were >10.0 and ∼0.23 μM, respectively, whereas those for malignant leukemia cells (Jurkat, U937, and HL-60), and HeLa cells were 0.07-0.08 and 0.17 μM, respectively. Under these conditions, the IC_50_ values of resveratrol against malignant leukemia cells and HeLa cells were 60.5-110.1 and 193.4 μM, respectively. These results demonstrate that malignant tumor cells are more sensitive to the antitumor activity of cis-3M-RES than normal T cells, and suggest that cis-3M-RES possesses at least 750-fold higher cytotoxicity against human tumors compared to the prototype resveratrol.

**Table 1:** Inhibitory effect of cis-3M-RES or RES on unstimulated human peripheral T cells, IL-2-dependent proliferation of PHA-stimulated peripheral T cells, and proliferation of tumor cells

Tumor and normal cells	Cis-3M-RES IC_50_ (μM)^*^	Resveratrol IC_50_ (μM)^*^
Unstimulated human peripheral T cells	>10.0	N.D
PHA-stimulated human peripheral T cells^**^	0.23	N.D
Human acute T cell leukemia Jurkat E6.1	0.07	110.1
Human acute T cell leukemia Jurkat (JT/Neo)	0.08	97.5
Human acute T cell leukemia Jurkat (JT/BCL-2)	>10.0	>250.0
Human monoblastoid U937	0.08	86.6
Human promyelocytic leukemia HL-60	0.08	60.5
Human cervical carcinoma HeLa	0.17	193.4

^*^ The IC_50_ value indicates a concentration of cis-3M-RES or resveratrol, which caused 50% reduction in cell viability based on the MTT assay. The cells were cultured with different concentrations of cis-3M-RES or resveratrol for 36 h and the final 4 h was incubated with MTT.

^**^ To induce IL-2-dependent proliferation of stimulated T cells, human peripheral T cells were stimulated with PHA (1.0 μg/ml) for 60 h, and then the stimulated T cells were harvested and incubated with various concentrations of cis-3M-RES at a density of 1 × 10^5^ cells/well as well as 25 U/ml of recombinant human IL-2 in 96-well plates.

Symbol: N.D, not determined.

## DISCUSSION

In this study, we first found that cis-3M-RES treatment of Jurkat T cells induces aberrant bipolar microtubule network formation and nuclear envelope breakdown, and prevents chromosomes congregation at the equatorial plate, all of which represent prometaphase arrest, before inducing the intrinsic mitochondria-dependent apoptotic pathway. This cis-3M-RES-induced prometaphase arrest was more apparently observed in JT/BCL-2 cells overexpressing BCL-2 because BCL-2 overexpression does not affect the prometaphase arrest but protects cells from undergoing apoptosis. Flow cytometric analysis with FITC-Annexin V and PI staining showed that necrosis was not involved in cis-3M-RES-induced apoptosis.

G_2_/M phase transition during cell cycle progression requires the activation of the CDK1/cyclin B complex through CDK1 phosphorylation at Thr-161 by the CAK and dephosphorylation at Thr-14 and Tyr-15 by phosphatase CDC25 [30, 31]. During progression from metaphase to anaphase, activated CDK1 undergoes inactivation because of the degradation of cyclin B by an anaphase-promoting complex. Sophisticated regulation of CDK1 activity is critical for cell cycle progression; however, prolonged activation of CDK1 because of MDAs-induced arrest at the mitotic spindle assembly checkpoint contributes to apoptosis induction [19, 42]. Failure of chromosome alignment on the equatorial plate in MDA-treated cells activates the mitotic spindle assembly checkpoint to prevent anaphase onset and maintains the CDK1/cyclin B complex in an active state by inhibiting the activity of the anaphase-promoting complex [26]. Because CDK1 is activated within 6 h and its activity is maintained until 18 h during the period of cis-3M-RES-induced G_2_/M arrest, as evidenced by the increase of cyclin B1 level and CDK1 phosphorylation at Thr-161 and dephosphorylation at Thr-14/Tyr-15 [30, 43], our results indicate the involvement of the mitotic spindle assembly checkpoint in the cis-3M-RES-induced mitotic arrest and apoptosis.

Comparative analysis of cis-3M-RES-induced apoptotic pathway between JT/Neo cells and JT/BCL-2 cells showed that cis-3M-RES-induced BAK activation, Δψm loss, caspase-9 and caspase-3 activation, and PARP cleavage, which were completely abrogated after BCL-2 overexpression, were crucial for apoptosis induction. Moreover, results of the present study showed that cis-3M-RES-induced mitotic arrest, CDK1 activation, BCL-2 phosphorylation at Ser-70, MCL-1 phosphorylation at Ser-159 and/or Thr-163, and BIM (BIM_EL_ and BIM_L_) phosphorylation occurred upstream of BCL-2-sensitive BAK activation and mitochondrial apoptotic events. Phosphorylation of anti-apoptotic BCL-2 proteins such as BCL-2 and MCL-1 by CDK1 has been reported to be crucial for the coupling of prolonged mitotic arrest with MDAs-induced apoptosis [18, 19, 33, 44].

Additionally, CDK1-dependent BIM phosphorylation is involved in apoptosis induced by MDAs such as taxol [45], 17α-estradiol [18], 2-methoxyestradiol [20], and nocodazole [19]. Phosphorylation induces conformational changes in BCL-2 that inactivate anti-apoptotic function and induce BAX and/or BAK activation to promote mitochondrial cytochrome *c* release [46, 47]. Phosphorylation of MCL-1 induces its degradation through ubiquitin-proteasome system, which increases the sensitivity of cells toward MDA-induced apoptosis [33]. Results of western blot analysis clearly detected BCL-2 phosphorylation at Ser-70, MCL-1 phosphorylation at Ser-159 and/or Thr-163, and BIM (BIM_EL_ and BIM_L_) phosphorylation in addition to cis-3M-RES-induced mitotic arrest and CDK1 activation. Notably, cis-3M-RES-induced phosphorylation of MCL-1 was accompanied with a remarkable reduction in MCL-1 expression level. Thus, the results of the present study as well as those of previous studies suggest that CDK1-mediated phosphorylation of BCL-2, MCL-1 and BIM is the most critical apoptotic events involved in cis-3M-RES-induced BAK activation, which leads to mitochondrial apoptosis.

To confirm whether mitotic arrest is required for CDK1-mediated BCL-2, MCL-1 and BIM phosphorylation, which induces mitochondrial apoptosis in cis-3M-RES-treated Jurkat T cells, we investigated the effect of enforced arrest at the G_1_/S border by APC treatment prevented cis-3M-RES-induced apoptotic events. APC treatment inhibits DNA polymerase α thus, inhibiting the entry of cells into the S phase [21]. Jurkat T cells treated concomitantly with 0.25 μM cis-3M-RES and 0.5 μM APC showed G_1_/S arrest but did not show the cis-3M-RES-induced apoptotic events, confirming that mitotic arrest is required for cis-3M-RES-induced apoptosis. Furthermore, results of immunofluorescence microscopic analysis showed that cis-3M-RES treatment induced prometaphase arrest in Jurkat T cells, as evidenced by the breakdown of the nuclear envelope and failure of the chromosome congregation at the metaphase plate because of the formation of aberrant bipolar microtubule network. Dependency of cis-3M-RES-induced apoptosis on impaired microtubule assembly and prometaphase arrest is very similar to that observed in our previous study, which showed that Jurkat T cells treated with MDAs such as 17α-estradiol, nocodazole, and 2-methoxyestradiol typically exhibited microtubule assembly impairment, prometaphase arrest, and apoptosis [18–20]. To examine whether the apoptogenic activity of cis-3M-RES was because of its action as an MDA, we examined the effect of cis-3M-RES on cellular microtubule polymerization in JT/BCL-2 cells in which BCL-2 overexpression did not affect microtubule damage and subsequent prometaphase arrest but prevented apoptosis induction. Treatment of JT/Neo cells with 0.1-1.0 μM cis-3M-RES decreased the proportion of intracellular polymeric tubulin similar to treatment with the well-known MDA nocodazole, indicating that cis-3M-RES-induced prometaphase arrest was provoked by the reduction in intracellular tubulin polymerization.

The critical requirement of CDK1 activity and caspase cascade activation for cis-3M-RES-induced apoptosis was further evaluated using the CDK1 inhibitor RO3306 [34] and the pan-caspase inhibitor z-VAD-fmk [35]. When the cis-3M-RES-induced histone H1 phosphorylation, which is catalyzed by the mitotic CDK1 [27], was inhibited to ∼40% of control level by 3 μM RO3306, the cis-3M-RES-induced Δψm loss and apoptotic sub-G_1_ cells were reduced to 52% and 45% of control levels, respectively. At the same time, phosphorylation of BCL-1, MCL-1 and BIM, caspase-9 and -3 activation, and PARP cleavage were significantly reduced. The cis-3M-RES-induced caspase cascade activation and apoptotic sub-G_1_ peak were almost completely abrogated by 30 μM z-VAD-fmk; however, the mitotic arrest, phosphorylation of BCL-2, MCL-1, and BIM, and Δψm loss were sustained. These results confirm that the cis-3M-RES-induced phosphorylation of BCL-2, MCL-1, and BIM, which led to mitochondria-dependent activation of the caspase cascade, was dependent on CDK1 activity and critically required for apoptosis induction.

To examine whether cis-3M-RES exerted different apoptogenic effect on tumor cells and normal cells, we compared the cytotoxicity of cis-3M-RES against various human leukemia cells (Jurkat, U937, and HL-60), human cervical carcinoma HeLa, and normal human T cells. Although the IC_50_ values of cis-3M-RES for malignant tumor cells tested were 0.07-0.17 μM, those for unstimulated human peripheral T cells and IL-2-dependent proliferation of PHA-stimulated peripheral T cells were >10.0 and ∼0.23 μM, respectively. These results indicate that normal human T cells were more refractory to cis-3M-RES-induced apoptotic activity than malignant tumor cells, which might permit a better application of cis-3M-RES in chemotherapy. Because cis-3M-RES-induced cytotoxicity against Jurkat T cells involved mitochondria-dependent activation of caspase cascade, it is likely that the better resistance of normal T cells than that of malignant Jurkat T cells to cis-3M-RES is due to the presence of poorly developed mitochondria and low levels of death signaling mediators in normal human T cells. In a recent study, trans-3,5,4′-trimethoxy-resveratrol (trans-3M-RES) at a concentration of 80 μM has been shown to inhibit cell growth though acting as a tubulin depolymerizing agent, which provokes multipolar spindles, mitotic arrest, and apoptosis in HeLa cells [48]. These previous and current data suggest that although trans-3M-RES and cis-3M-RES may cause in common microtubule damage-mediated antitumor activity, the potency of cis-3M-RES is dramatically higher than that of trans-3M-RES.

In conclusion, our results showed that the cis-3M-RES-induced apoptotic pathway in Jurkat T cells was proceeded by the reduction of microtubule polymerization, which impaired the mitotic spindle network, and induction of prometaphase arrest; prolonged CDK1 activation; and BCL-2, MCL-1, and BIM phosphorylation, which increased the susceptibility of these cells to mitochondria-dependent apoptosis by triggering BAK activation, loss of Δψm, and caspase cascade activation. These results are useful for evaluating the potency of cis-3M-RES as an antitumor agent.

## MATERIALS AND METHODS

### Reagents, antibodies, and cells

Cis-trimethoxy resveratrol (cis-3,5,4′-trimethoxy-resveratrol, cis-3M-RES, 185 mM/ethanol) was purchased from Cayman Chemical (Ann Arbor, Michigan, USA). Resveratrol (3,5,4′-trihydroxystilbene), APC, DiOC_6_, and DAPI were purchased from Sigma Chemical (St. Louis, MO, USA). An ECL western blotting kit was purchased from Amersham (Arlington Heights, IL, USA), and the Immobilon-P membrane was obtained from Millipore Corporation (Bedford, MA, USA). Anti-caspase-3 antibody was purchased from Pharmingen (San Diego, CA, USA), and anti-PARP, anti-BIM, anti-BCL-2, anti-MCL-1, anti-CDK1, anti-cyclin B1, anti-p-histone H3 (Ser-10), anti-histone H3, anti-lamin B, and anti-β-actin antibodies were purchased from Santa Cruz Biotechnology (Santa Cruz, CA, USA). Anti-caspase-9, anti-p-CDK1 (Tyr-15), anti-p-CDK1 (Thr-161), anti-p-CDC25C (Thr-48), anti-CDC25C, anti-p-BCL-2 (Ser-70), anti-p-MCL-1 (Ser-159/Thr-163), and anti-α-tubulin antibodies were purchased from Cell Signaling Technology (Beverly, MA, USA). Anti-GAPDH antibody was purchased from Thermo Scientific (Rockford, IL, USA). Anti-p-histone H1 was purchased from Upstate Biotechnology (Lake Placid, NY, USA) and anti-BAK (Ab-1) was purchased from Calbiochem (San Diego, CA, USA). Human acute leukemia wild-type Jurkat T cell clone A3, Fas-associated death domain (FADD)-deficient Jurkat T cell clone I2.1, caspase-8-deficient Jurkat T cell clone I9.2, Jurkat T cell clone E6.1, U937, HL-60, and human cervical carcinoma HeLa cells were purchased from ATCC (Manassas, VA, USA). These cells were maintained in RPMI 1640 medium (Hyclone, Gaithersburg, MD, USA) containing 10% FBS, 20 mM Hepes (pH 7.0), 50 μM 2-mercaptoethanol, and 100 μg/ml gentamycin. HeLa cells were maintained in DMEM (Hyclone) supplemented with 10% FBS, 20 mM Hepes (pH 7.0), and 100 μg/ml gentamycin. Jurkat T cell clone, stably transfected with an empty vector (JT/Neo) or with the *BCL-2* expression vector (JT/BCL-2) was kindly provided by Dr. Dennis Taub (Gerontology Research Center, NIA/NIH, Baltimore, MD, USA). Both JT/Neo cells and JT/BCL-2 cells were maintained in RPMI 1640 medium containing 10% FBS, 20 mM Hepes (pH 7.0), 50 μM β-mercaptoethanol, 100 μg/ml gentamicin, and 400 μg/ml G418. JT/BCL-2 cells overexpressing BCL-2 and JT/Neo cells were identified by performing western blot analysis. These stable clones were kept in culture for no more than 3 months before the studies, and used in our several previous investigations including a recent study [20].

### Cytotoxicity assay

The cytotoxic effect of cis-3M-RES on unstimulated human peripheral T cells, PHA-stimulated peripheral T cells, human leukemia cells (Jurkat, U937, and HL-60), and human cervical carcinoma HeLa cells was analyzed by MTT assay. Briefly, cells (5 × 10^4^ cells/well for leukemia cells, 2.5 × 10^3^ cells/well for HeLa cells, 2 × 10^5^ cells/well for unstimulated human peripheral T cells, and 1 × 10^5^ cells/well for PHA-stimulated peripheral T cells) were added to the serial dilution of cis-3M-RES in 96-well plates. At 32 h after incubation, 50 μl of MTT solution (1.1 mg/ml) was added to each well and incubated for an additional 4 h. After centrifugation, the supernatant was removed from each well, and then 150 μl of DMSO was added to dissolve the formazan crystals produced from MTT. OD values of the solutions were measured at 540 nm by a plate reader.

### DNA fragmentation analysis

Apoptotic DNA fragmentation induced in Jurkat T cells treated with cis-3M-RES was determined by performing Triton X-100 lysis with 1.2% agarose gel electrophoresis as previously described [49].

### Flow cytometric analysis

Flow cytometric analysis to measure the cell cycle state of Jurkat T cells treated with cis-3M-RES was performed on a FACS Calibur (BD Sciences, San Jose, CA, USA) as described elsewhere [49]. The extent of necrosis was detected using an FITC-Annexin V apoptosis kit as described previously [49]. Changes in the mitochondrial membrane potential (Δψm) following cis-3M-RES treatment were measured after staining with DiOC_6_ [22, 50]. Activation of BAK in Jurkat T cells following cis-3M-RES treatment was measured as previously described [23].

### Immunofluorescence microscopy

Immunostaining of Jurkat T cells treated with cis-3M-RES was performed as previously described [18].

### Preparation of cell lysates and western blot analysis

Cell lysates were prepared by suspending 5 × 10^6^ Jurkat T cells in 300 μl of lysis buffer as described elsewhere [49]. Equivalent amounts of protein lysate (20 μg) were electrophoresed on a 4-12% NuPAGE gradient gel (Invitrogen/Novex, Carlsbad, CA, USA) and then electrotransferred to an Immobilon-P membrane. Protein detection was performed using an ECL western blot kit according to the manufacturer's instructions. Densitometry was performed using ImageQuant TL software (Amersham, Arlington Heights, IL, USA). The arbitrary densitometric units for each protein of interest were normalized using those for GAPDH or β-actin.

### Extraction of monomeric and polymeric tubulin

Monomeric tubulin fraction was prepared by extracting cells in a monomeric extraction buffer (20 mM PIPES, 0.14 M NaCl, 1 mM MgCl_2_, 1 mM EGTA, 0.5% NP-40, and 0.5 mM PMSF, pH 6.8) as described previously [20] with some modifications. After centrifugation at 13,000 × g for 10 min at room temperature, the NP-40-soluble fraction containing monomeric tubulin was collected. The polymeric tubulin fraction was prepared by disrupting the remaining insoluble material in RIPA buffer (0.15 M NaCl, 1% deoxycholate, 1% NP-40, 0.1% SDS, and 10 mM Tris, pH 7.4) followed by centrifugation. An equivalent amount of each fraction sample was electrophoresed on a 4-12 % NuPAGE gradient gel. Western blot analysis for α-tubulin was performed as described in the Materials and methods section.

### Isolation and activation of human peripheral T cells

To prepare human peripheral blood mononuclear cells (PBMC), heparinized blood obtained from healthy laboratory personnel by venipuncture was centrifuged at 800×g for 20 min over Ficoll (Sigma Chemical, St. Louis, MO, USA), according to the manufacturer's instructions. This protocol was approved by the Ethics Committee of Kyungpook National University, Daegu, Korea. Informed written consent was obtained from the participants. T cells were isolated from PBMCs by using a human T cell enrichment column kit (R&D Systems, Minneapolis, MN, USA). The isolated peripheral T cells were activated by incubating 2 × 10^6^ cells/ml with 1.0 μg/ml PHA for 60 h. To induce IL-2-dependent T cell proliferation, PHA-stimulated T cells (1 × 10^5^/well) were cultured with 25 units (U) of human recombinant IL-2 in 96-well plates.

### Statistical analysis

Unless otherwise indicated, each result in this study is a representative of at least three separate experiments. Values are expressed as the means ± standard deviation (SD) of these experiments. Statistical significance was calculated using Student′s *t*-test. *P* values of <0.05 were considered statistically significant.
